# Broadband Tunability of Polarization-Insensitive Absorber Based on Frequency Selective Surface

**DOI:** 10.1038/srep23081

**Published:** 2016-03-17

**Authors:** Han Wang, Peng Kong, Wentao Cheng, Wenzong Bao, Xiaowei Yu, Ling Miao, Jianjun Jiang

**Affiliations:** 1 School of Optical and Electronic Information, Huazhong University of Science and Technology, Wuhan, 430074, China

## Abstract

An innovative tunable and polarization-insensitive 1.6–8 GHz frequency selective surface (FSS) absorber was investigated in this study. The proposed FSS, which is in 4-axial symmetrical form, includes a novel array of PIN diodes with biasing lines including inductors. A gradually reduced equivalent resistor of PIN diodes can be achieved with increasing DC voltage, which characterizes tunable, multi-resonance absorption peaks. Via this simplified design, small value resistor and equivalent capacitance of the gap between patterns can improve the absorber’s performance in low frequencies; an active tunable absorber can be realized in a broad frequency range by employing adjustable devices. Changing the working state of the PIN diode allows the user to obtain strong absorption within the desired frequency. We analyzed the performance of the proposed absorber and found that it indeed shows very favorable absorption performance in low frequency (−10 dB in 1.6**−**4.3 GHz) and wideband (−8 dB in 4.3**−**5.4 GHz and −10 dB in 5.4**−**8.0 GHz) conditions. Calculation and simulation results also illustrated that the absorber is entirely polarization-insensitive.

Electromagnetic (EM) wave absorbers are invaluable components of stealth systems. In recent years, several artificial materials have been used to fabricate broadband EM wave absorbers^1^,^2^,^3^. One of these, the frequency selective surface (FSS) absorber, is comprised of periodic resonators and dielectric layers^4^,^5^. Multi-ring patterns, fractal patterns, and varied periodic patterns have been reported to expand the absorptive bandwidth of FSS absorbers due to their multi-resonance characteristics^3^,^6^,^7^. Manipulating the resonant state also allows the designer to control broadband properties; the tunable FSS (or “active” FSS) absorber allows the resonant state to be tuned and the working band adjusted as necessary to absorb EM waves at the desired frequency^8^. When designing a tunable absorber, the focus is on the entire adjustable bandwidth not the broadband from a single resistor value. Accordingly, the absorber can achieve strong absorption at different frequency bands via adjustable work state, and has an equivalent broadband with different resonant states. In a previous study conducted in our laboratory, we demonstrated an absorber cell pattern with gradational edges to have multi-resonance characteristics. Assisted by active devices, this multi-resonant pattern can be used as the unit cell resonator of a broadband tunable absorber applied in 1–5 GHz^9^. Active parameters and multi-resonance patterns together benefit broadband absorption, creating an FSS absorber with wider and tunable absorption broadband (including low frequencies,) to 1.6 GHz.

Polarization-insensitive characteristics are important in many EM applications, including EM wave absorbers. Several previous researchers have successfully realized polarization-insensitive absorbers (PIA)^10^,^11^,^12^. Conventional methods to design PIAs include using highly symmetrical unit cell patterns such as 90° rotational symmetry^10^, 4-axial symmetry^11^, or higher symmetry^12^. In these highly symmetrical configurations, periodic resonators are able to resonate similarly at different polarized incident waves. This design strategy has been also utilized to accomplish tunable PIA. Zhu *et al*.^13^, for example, demonstrated a controllable EM wave reflector/absorber for different polarizations involving orthogonally coupled patterns coupled with diodes. Zhao *et al*.^14^ realized an electrically controlled metamaterial absorber using patterns placed orthogonally to form 4-axial symmetrical unit cells. At different polarized incident waves, the resonant portion of the symmetrical pattern lies along different directions, and because the bias networks of tunable PIAs must be designed to avoid shorting the resonant parts at different polarized incident waves, tunable PIA are considerably more difficult to design than passive PIA.

In this study, we realized a broadband-tunable PIA based on FSS. Patterns with gradational edges were chosen as resonant units, and were improved to 4-axial symmetrical patterns to ensure polarization-insensitive characteristics. PIN diodes were embedded between adjacent resonant units to allow the absorptive frequency to be electrically controlled. The experimental results verify the tunable and polarization-insensitive characteristics of the designed broadband FSS absorber, ranging from 1.6 to 4.3 GHz and 5.4 to 8 GHz with reflectivity below −10 dB, and 4.3 to 5.4 GHz with reflectivity below −8 dB.

## Design and Simulation

**Figure 1:**
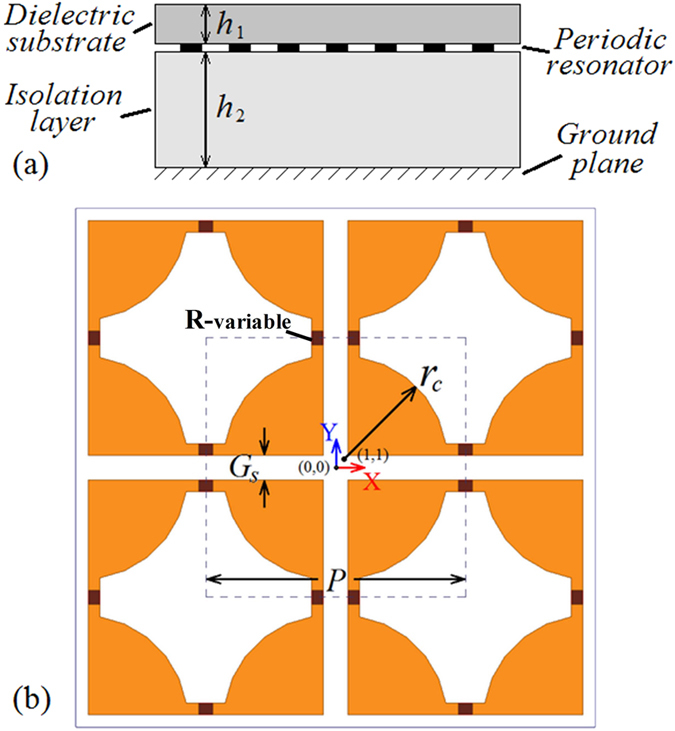
Schematic diagram of absorber (metal parts marked in yellow). (**a**) Topology of the FSS structure; (**b**) unit cell geometry. Geometrical parameters are P = 36 mm, *r*_c_ = 14.5 mm, *G*s = 3.4 mm.

The proposed structure of the broadband-tunable FSS PIA is based on one of our previous absorber designs^9^, which we modified into a 4-axial symmetrical pattern in order to take polarization into account. The PIA consists of a dielectric substrate on the top layer, an array of the pattern with gradational edges below the substrate, and an isolation layer on top of the metallic ground plane, as shown as Fig. 1(a). The substrate layer material is FR4 with a permittivity of 4.4, loss tangent of 0.02, and thickness marked as *h*_1_. The primary effect of FR4 in the design is to support metallic patterns. A honeycomb support layer is used as an isolation layer (thickness labeled *h*_2_,) selected for its high strength and low density. The PIA is in planar-periodic structure, and the period of the unit cell is 36 mm (*P*). The unit cell resonator is comprised of metallic patterns and variable resistors named *R*-variables, as shown in Fig. 1(b). The metallic patterns consist of four quarter circles and plates form the gaps (*G*_*s*_).

**Figure 2:**
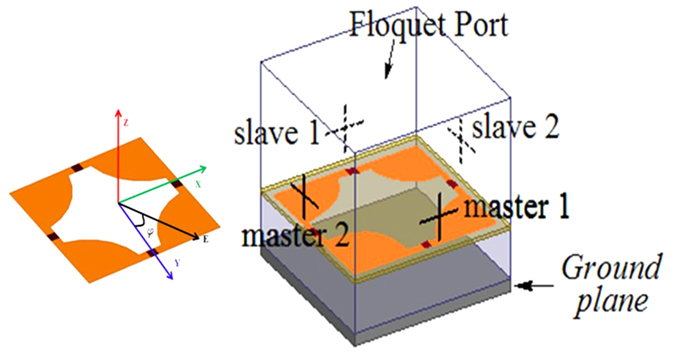
Simulation model of tunable PIA.

The unit cell pattern of the FSS was modeled in the High Frequency Structure Simulator (HFSS). Finite element method (FEM) was used to simulate the electromagnetic properties of the FSS structure, and the metallic patch of the pattern and the ground plane were modeled by the perfect electric conductor (PEC) boundary. The master-slave boundary condition was adopted in the side faces. A Floquet port was fixed on the top boundary along the Z direction, as a normal incidence wave, and the angle between E vector (electric field) and the Y axis is **ϕ** in the plane of the array. The simulation model is shown in the inset of Fig. 2.

All parameters above were extracted via GA optimization. The two dimension parameters (*r*_*c*_, *G*_*s*_) and two structure parameters (*h*_1_, *h*_2_) were optimized based on the multi-value setting of the resistors (*R*-variables). The dimension of the plates was then *r*_*c*_ = 14.5 mm, the width of gaps was *G*_*s*_ = 3.4 mm, and the total thickness of *h*_1_ and *h*_2_ was 11.9 mm. As resistor value changed, the FSS absorber showed different absorption performance – to this effect, our prototype is an absorption-peak-position-adjustable FSS absorber.

During simulation, the resistor was respectively set to 50 Ω, 100 Ω, 150 Ω, 200 Ω, 350 Ω, and 500 Ω. The envelope curve was drawn according to the reflectivity performance of each resistor value. Values below 50 Ω or above 500 Ω do not benefit the envelope. We found it better to consider the paralleled parasitic capacitance (C_T_) in the simulation, because we used the PIN diode BAP 50–03 and took measurements according to voltage. Parasitic parameters were extracted by way of fitting: C_T_ = 0.05 pF. The polarization independence of the proposed structure was demonstrated by simulating different polarization states (0°, 30°, 45^o^, 60°, and 90°)

**Figure 3:**
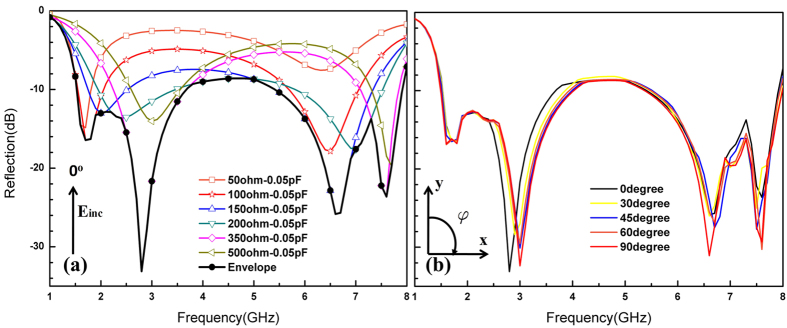
Simulated reflectivity coefficients. (**a**) Simulation reflectivity of FSS with different resistors and envelope of 0°; (**b**) comparison between envelopes for different polarization angles **ϕ**.

The simulation results at angle **ϕ** of 0° under different resistor values and envelope curves are shown in Fig. 3(a). The reflectivity of the envelope is below −8 dB in 1.5**−**8 GHz. Table 1 lists the tunability percentages of the proposed broadband absorber with various resistors, where absorption peak positions and corresponding reflectivity values change under different resistor values, contributing to the PIA’s unitary performance. Figure 3(b) shows the plotted envelopes for the pattern illuminated with a linearly polarized source at rotation angles **ϕ** of 0°, 30°, 45°, 60°, and 90° at normal incidence; this information was used in accordance with the envelopes.

## Experiment and Discussion

**Figure 4:**
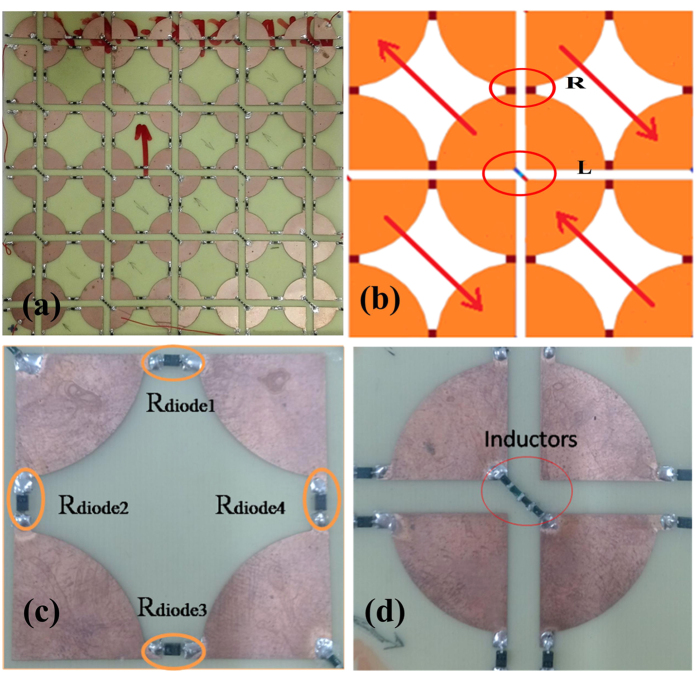
Photograph of FSS prototype. (**a**) Sample of tunable polarization-insensitive broadband FSS; (**b**) diagram of current flows; (**c**,**d**) magnified photos of the pattern.

We fabricated a 180 mm × 180 mm sample of the broadband tunable, polarization-insensitive absorber as shown in Fig. 4(a). Figure 4(c,d) show actual images of the resistors (R) and inductors (L) in Fig. 4(b). A PIN diode BAP50-03 served as the variable resistor, as shown in Fig. 4(c). The PIN diode was modeled as a parallel combination of R_p_ and C_T_, which was determined during simulation. At frequencies above about 10 MHz and up to several GHz, the equivalent circuit proved to be a good approximation to typical resistance with value controlled by a DC or low-frequency control current. PIN diodes work as resistors with high resistance in the microwave frequency band at small forward bias voltages, and with low resistance at large forward voltages. The BAP 50–03 tunable device was supplied voltage in a row, so it sufficiently replaced the variable resistor used for simulation during experimentation.

In AFSS designs, it is challenging to distribute control or bias lines appropriately. Compared to the pattern used in our previous study^9^, this pattern did not easily provide voltage to the PIN diodes in parallel, so we had to create a new design. As shown in Fig. 4(d), inductors were used instead of metal conducting wire, and Fig. 4(b) shows how we powered up the PIN diodes and current flow. All PIN diodes were connected in series instead of parallel to ensure the diodes in each period obtained the same voltage, conforming to the simulation. We chose inductors (SIMID 0603 series from EPCOS, specifically,) to place between the two metallic plates in the same period that would link up PIN diodes and achieve the required isolation without impacting the PIA’s operation and performance^15^. To attain 1–8 GHz isolation, considering the distance of the two plates is 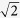
*G*_s_ (4.5 mm), we found it better to weld four inductors as control lines (4.7 nH, 10 nH, 22 nH, and 68 nH.)

**Figure 5:**
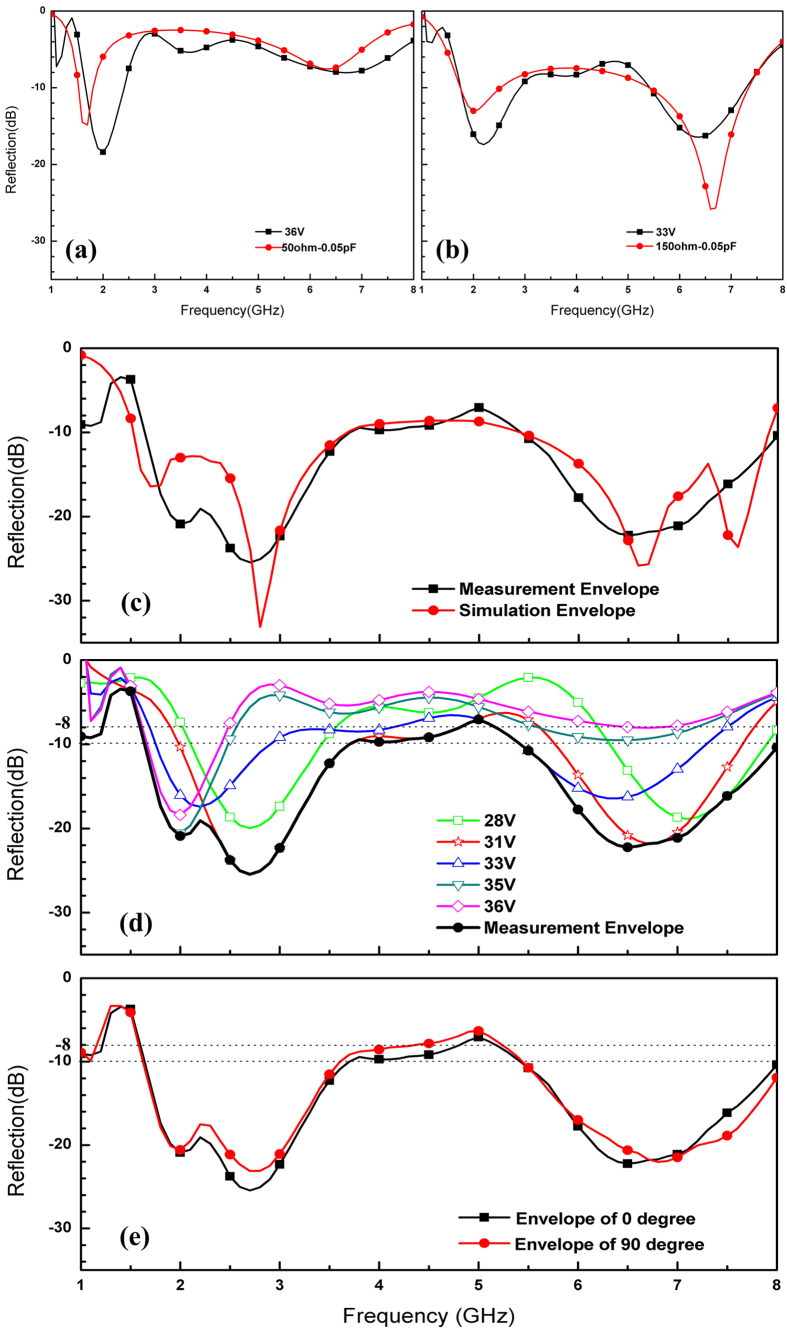
Reflectivity measurement results. (**a**,**b**) Comparison between simulation and measurement in different working states; (**c**) envelope comparison between simulation and measurement; (**d**) reflectivity with different voltages and envelope of 0°; (**e**) measurement results at 0° and 90°.

The microwave reflectivity of the sample was examined by arch test method with two broadband double-ridged horn antennas and a vector network analyzer (Agilent 8720ES). Results for different polarization states were obtained by rotating the pattern in a certain angle for the unchanged incidence wave, at which point the electric field was located along different directions of the pattern. The experimental results and comparisons of different angles are plotted in Fig. 5, including the performance of the tunable absorber in different voltages and their envelopes. Comparisons between experimentation and simulation are shown in Fig. 5(a–c). Changes in reflectivity as a function of frequency were similar in the same states between the experiment and the simulation.

During the experiment, the absorber was measured at angles of 0° and 90°; comparisons between the two angles are shown in Fig. 5(e). We found that the envelopes of reflectivity at 0° and 90° almost coincided, and that control biases and adjustable devices did not affect the polarization–insensitive characteristics of the structure. The trends and rules observed in the experiment showed close agreement with those obtained through simulation, demonstrating that the simulated results are accurate, the proposed manner of enhancing polarization characteristics is feasible, and the tunable absorber we propose has favorable polarization stability.

Figure 5(d) shows where the tunable FSS had different absorption peaks corresponding to different bias voltages, (as expected based on the simulation results.) Shown as the black curve in Fig. 5(d), the envelope of all reflectivity clearly showed multiple absorption peaks; PIN diodes clearly contributed to shifting resonant frequency. Similar to the simulation results, reflectivity decreased 10 dB from 1.6 GHz to 4.3 GHz and 5.4 GHz to 8 GHz. From 4.3 GHz to 5.4 GHz, reflectivity values almost all fell between −8 dB and −10 dB. To this effect, our primary design goals – polarization insensitivity and broadband tenability in the FSS – were successfully realized.

By comparison, trends and amounts of absorption peaks for every curve between the simulation and experiment were similar. Both envelopes showed multiple strong absorption points, shown as Figs 3(a) and 5(d). The experiment results showed favorable performance in low frequencies and wider bands with 3 GHz with one tunable device compared to our previous design, which contained two tunable devices^9^.

### Tunable Absorber Physical Mechanism

**Figure 6:**
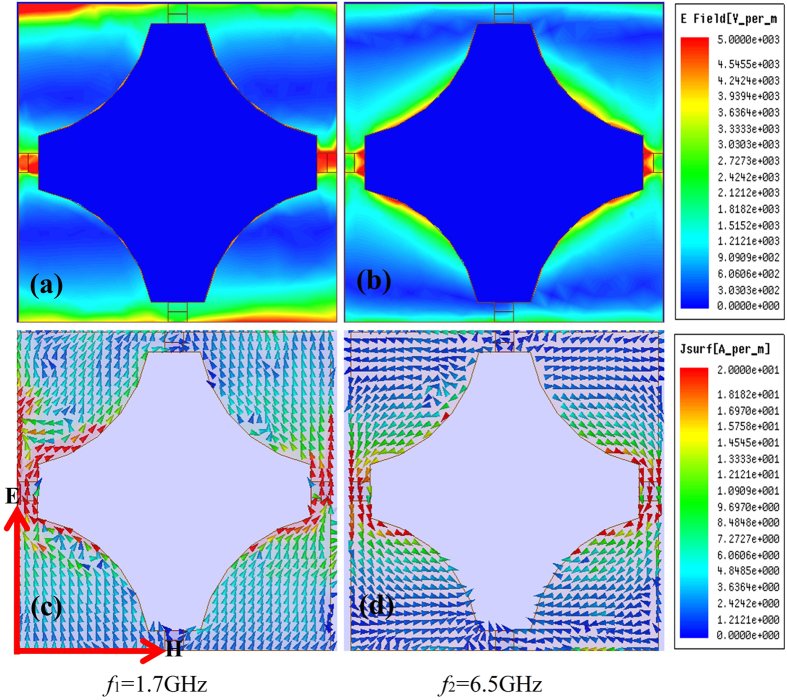
Electric field and surface current field at two resonance peaks with 100 Ω. (**a**,**c**) First absorption peak, *f*_1_ = 1.7 GHz; (**b**,**d**) second absorption peak, *f*_2_ = 6.5 GHz.

There were two absorption peaks for each state in both the experiment and the simulation: one at low frequency and one at high frequency. Figure 6 shows the electric field (E) and current vector fields of the two absorption peaks with 100 Ω. The strong electric field distributed at different resonant states, as well as the opposite current directions, suggest that the resonances were generated from different parts of the pattern. Compared to other parts, there was a strong electric field around gaps and resistors as shown in Fig. 6(a); the gaps provided capacitance along the direction of electric field. The first absorption peak of every resistor was a result of interactions among gap capacitance and resistors. The second resonance peak, as shown in Fig. 6(b), primarily occurred in the vicinity of gradational edges and resistors.

**Figure 7:**
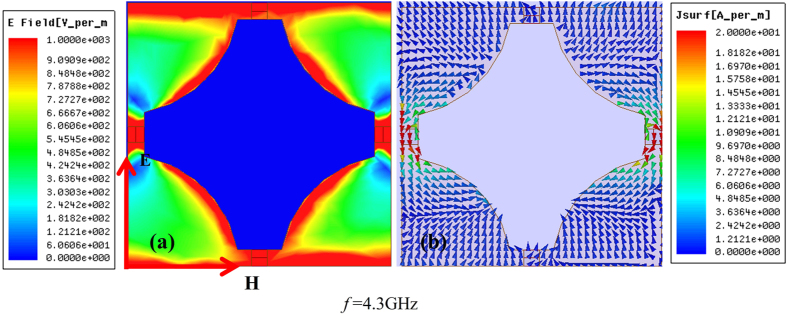
Electric field and surface current field at 4.3 GHz with 100 Ω.

In fact, gaps and gradational edges both affected resonance within all frequency bands. Figure 7(a) depicts the electric field with 100 Ω at middle frequency 4.3 GHz. Electric fields distributed both around gaps and gradational edges were apparent when changing the field’s magnitude to 1000 V/m. (Note the electric field magnitudes in Figs 6 and 7 are different.) The two kinds of resonance effects were both too weak to produce an absorption peak (see measurements at 1.7 GHz and 6.5 GHz,) and belong to the same order of magnitude. As such, it was easy to observe the two distinct types of resonance mechanism homogeneously in one graph. Though these two mechanisms should be clear in Fig. 6(a,b), only one of them is the primary factor within the frequency band located at the resonance point, so only one resonance mechanism is observable in the figure.

As shown in Figs 6 and 7, there was a strong electric field around resistors and capacitance (i.e., PIN diodes,) so there were intense currents along the direction of the E-vector due to sizeable difference in electric intensity, visible in Fig. 6(c,d). The energy of the incident microwave was translated into heat energy by the adjustable device PIN diode under the effects of induced current, which was the primary cause of energy loss around the resonance peak. 4.3 GHz was not the resonance peak, but still decreased by 5.4 dB, where energy was still mostly consumed by the PIN diodes. To this effect, adjustable devices like the one proposed here contribute substantially to absorbing performance.

Of course, careful design of the gaps and gradational edges and the use of the adjustable device are crucial to the success of the structure overall. When R-variable increases, the change in resistance affects the distribution of the electric field. The resonance peaks also moves backwards gradually as the electric field distribution changes. The dimensions of *r*c and *G*s are also significant, as they determine performance. Specifically, changing the value of *r*c and *G*s causes the position of the resonant frequency to move. Further research should focus on utilizing this pattern to design a structure that performs well at even lower frequencies and wider bands.

### Oblique Incidence Behavior of Structures

**Figure 8:**
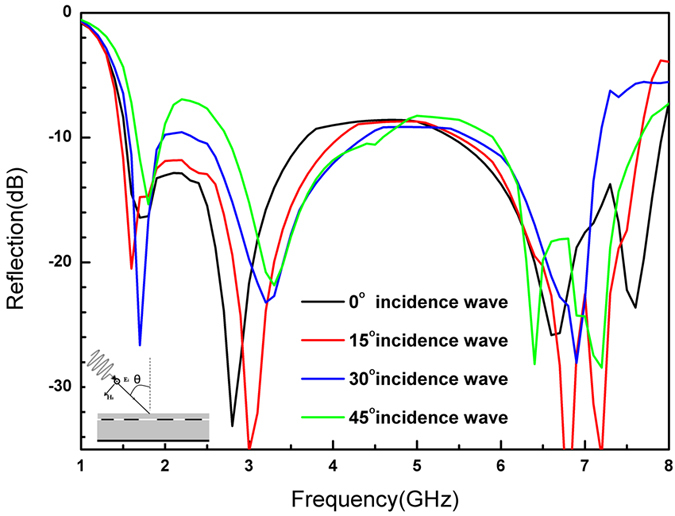
Comparison for TE incidence at different angles (1) 0° (2) 15° (3) 30° (4) 45°.

The proposed structure demonstrated favorable performance at normal incidence, but the situation would be totally different if the absorber had oblique angles of incidence. We simulated oblique incidence behavior of structures for TE mode at incidence angles of 0°–45° in 15° increments as shown in Fig. 8 to explore this phenomenon, and found that the positions of absorption peaks moved to high frequency as the incidence angle increased. At the same time, the envelope curves become narrower and the depth shallower. The structure was still adjustable in a wide band and stable with different incidence angles, however. Further research on the incidence behavior of the proposed structure would be useful for optimizing the design.

## Conclusion

In this study, a 4-axial symmetrical pattern was developed based on a previously proposed pattern and applied to a sample finite 5 × 5 unit cell FSS structure. Due to structural symmetry, the absorber we propose proved insensitive to the polarization direction of the incident EM wave. As we stated in our previous study, multi-resonances exit in patterns with gradational edges^9^. Using several inductors instead of metallic biasing lines can successfully isolate 1–8 GHz from the alternating signal without impacting the PIA’s operation and performance. The supplied voltages allow the diodes to function as tunable devices, generating multi-resonances with the patterns. We found two absorption peaks at every voltage tested, showing that the proposed absorber has a wide frequency band. Due to the gap capacitances of the designed pattern, the structure also has an advantage at low frequencies. Although there were notable differences, the results between the experiment and simulation that we conducted were sufficiently similar. The envelopes reached a target of −10 dB bandwidth of 5.3 GHz (1.6**−**4.3 GHz, 5.4**−**8 GHz), and −8 dB bandwidth of 6.4 GHz (1.6**−**8 GHz). Experiment results verified that the designed tunable absorber exerts favorable absorption effect on all polarizations in a wide band, and further, according to the simulated oblique incidence behavior, the absorber may show very good performance under large scan angles (usually up to 45°) though this will need to be confirmed through further research. Evidenced by its performance, the absorber designed in this study shows attractive potential application in stealth technology and EM interference shielding technology.

## Additional Information

**How to cite this article**: Wang, H. *et al*. Broadband Tunability of Polarization-Insensitive Absorber Based on Frequency Selective Surface. *Sci. Rep*. **6**, 23081; doi: 10.1038/srep23081 (2016).

## Figures and Tables

**Table 1:** Percentages of tunability of proposed broadband absorber for various resistors.

Resistance change(Ω)	Frequency change (GHz)	Percentage change	|Reflectivity| change (dB)	Percentage change
50–100	1.700–1.700	0.000%	14.837–16.421	10.676%
	6.300–6.500	3.175%	7.551–17.843	136.300%
50–150	1.700–2.000	17.647%	14.837–13.019	−12.253%
	6.300–6.600	4.762%	7.551–25.831	242.087%
50–200	1.700–2.400	41.18%	14.837–13.646	–8.027%
	6.300–7.000	11.111%	7.551–17.594	133.002%
50–350	1.700–2.800	64.706%	14.837–33.112	123.172%
	6.300–7.600	20.635%	7.551–23.627	212.899%
50–500	1.700–3.000	76.471%	14.837–14.009	−5.581%
	6.300–7.700	22.222%	7.551–19.727	161.250%
